# Use of RNA-seq to identify genes encoding cytokines and chemokines activated following uptake and processing a candidate peptide vaccine developed against *Mycobacterium avium* subsp. *paratuberculosis*

**DOI:** 10.29374/2527-2179.bjvm002723

**Published:** 2024-01-16

**Authors:** Michelle Athena Decourcey, William Charles Davis, Cleverson de Souza

**Affiliations:** 1 Veterinarian, Veterinary Medical Diagnostic Laboratory, University of Missouri College of Veterinary Medicine, Columbia, CO, USA; 2 Veterinarian, PhD, Department of Veterinary Microbiology and Pathology, Washington State University, Pullman, WA, USA; 3 Veterinarian Department of Comparative, Diagnostic & Population Medicine, University of Florida, Gainesville, FL, USA

**Keywords:** Mycobacterium avium subspecies paratuberculosis, monocyte-derived macrophage, bovine, peptide vaccine, RNA-seq, Mycobacterium avium subespécie paratuberculosis, macrófago derivado de monócitos, bovino, vacina peptídica, RNA-seq

## Abstract

Analysis of the primary and recall responses to a membrane molecule (MMP), encoded by MAP2121c demonstrated that tri-directional signaling between the antigen-presenting cell (APC), CD4 and CD8 is essential for eliciting a CD8 cytotoxic T cell (CTL) response against Mycobacterium avium subsp. paratuberculosis. As reported here, RNA-sequencing was used to initiate the characterization of the signaling pathways involved in eliciting the development of CD8 CTL, starting with the characterization of the activation status of genes in monocyte-derived macrophages (MoMΦ) following uptake and processing MMP for the presentation of antigenic epitopes to CD4 and CD8 T cells. Activation status was compared with the uptake and processing of LPS, a nonspecific stimulator of macrophages. 1609 genes were identified that were upregulated, and 1277 were downregulated three hours after uptake and processing MMP. No significant difference was observed in the cytokine genes selected for analysis of the signaling that must occur between APC, CD4, and CD8 for the development of CTL. The initial observations indicate screening of the transcriptome should include genes involved in signaling between APC and CD4, and CD8 regardless of their activation status. Four genes of interest in this study, IL12A, IL12B, IL15, and IL23A, were not significantly different from control values. The initial studies also indicate MoMΦ can be included with dendritic cells and monocyte-derived dendritic cells for further analysis of the tri-directional signaling required for the development of CTL.

## Resumo

A análise das respostas primárias e de recall a uma molécula de membrana (MMP), codificada por MAP2121c demonstrou que a sinalização tridirecional entre a célula apresentadora de antígeno (APC), CD4 e CD8 é essencial para provocar uma resposta de células T citotóxicas CD8 (CTL) contra *Mycobacterium avium* subsp. *paratuberculose*. Conforme relatado aqui, o sequenciamento de RNA foi usado para iniciar a caracterização das vias de sinalização envolvidas na indução do desenvolvimento de CTL CD8, começando com a caracterização do status de ativação de genes em macrófagos derivados de monócitos (MoMΦ) após captação e processamento de MMP para a apresentação de epítopos antigênicos às células T CD4 e CD8. O status de ativação foi comparado com a captação e processamento de LPS, um estimulador inespecífico de macrófagos. Foram identificados 1.609 genes que foram regulados positivamente e 1.277 foram regulados negativamente três horas após a captação e processamento de MMP. Nenhuma diferença significativa foi observada nos genes de citocinas selecionados para análise da sinalização que deve ocorrer entre APC, CD4 e CD8 para o desenvolvimento de CTL. As observações iniciais indicam que o rastreio do transcriptoma deve incluir genes envolvidos na sinalização entre APC e CD4 e CD8, independentemente do seu estado de activação. Quatro genes de interesse neste estudo, IL12A, IL12B, IL15 e IL23A, não foram significativamente diferentes dos valores de controle. Os estudos iniciais também indicam que o MoMΦ pode ser incluído com células dendríticas e células dendríticas derivadas de monócitos para análise adicional da sinalização tridirecional necessária para o desenvolvimento de CTL.

## Introduction

*Mycobacterium avium* subsp. *paratuberculosis* (*Map*) is a prototypic member of a lineage of bacteria that emerged before the dawn of civilization (Bachmann et al., 2020). *Map* was identified as the causative agent of paratuberculosis by Johne and Frothingham at the end of the nineteenth century (Johne’s disease in cattle) (Johne & Frothingham, 1895). Similar to other mycobacterial pathogens, infection with *Map* leads to development of an immune response that controls but does not clear infection. A persistent infection develops that is under immune control. This type of immunity may be sufficient to prevent clinical disease for the lifetime of the host species or break down. For cattle, protective immunity breaks down two to three years following exposure. Because of the lack of methods to detect infected animals during this latent period of infection, latently infected animals have been introduced into *Map* free herds in the US and other countries. In recognition of the impact of paratuberculosis on the livestock industry and human health, national and international programs have been underway to elucidate the mechanisms used by *Map* and other mycobacterial pathogens to evade immune elimination and develop vaccines. The sequencing of the genomes of *Map*, *Mycobacterium tuberculosis* and *M. bovis*, and methods to selectively delete genes has made it possible to determine the effect of gene deletion on the capacity of mycobacterial pathogens to evade immune elimination. It has also made it possible to identify expressed gene products for potential use in peptide-based vaccines. We have focused on the use of *Map* as a prototypic mycobacterium to characterize the immune response to pathogenic mycobacteria and to determine the effect of gene deletion on capacity to persist in vivo. We have used cattle as the model species for study of the immune response and development of candidate vaccines. The initial studies demonstrated exposure leads to infection of all animals under experimental conditions and development of humoral and cellular immunity (Davis et al., 2021; Koo et al., 2004). Studies on the stringent response (ability to survive in unfavorable environments), to the H37Rv strain of *Mycobacterium tuberculosis* (*Mtb*) demonstrated deletion of *relA,* a gene involved in regulating the stringent response (reviewed in (Kundra et al., 2020)), abrogated its ability to survive in a mouse model (Dahl et al., 2003). Follow up studies with *Map* yielded identical results suggesting *relA* might be Achilles’ heel for mycobacteria (Abdellrazeq et al., 2020b). A deletion mutant was unable to establish a persistent infection in cattle and goats (Park et al., 2011). Vaccination led to development of CD8 cytotoxic T cells (CTL) that killed *Map* present in macrophage target cells. Further studies demonstrated the target of the CTL was a 35 kD cell major membrane protein (MMP) (Abdellrazeq et al., 2018).

The most recent studies have focused on analysis of the immune response to MMP using an ex vivo tissue culture platform. The studies have shown that concurrent tri-directional signaling must occur between APC and CD4 and CD8 T cells for development of CD8 CTLs (Abdellrazeq et al., 2020a). The objective of the present investigation was to conduct a pilot study with RNA-sequencing (RNA-seq) to determine if monocyte-derived macrophages (MoMΦ) could be used to initiate studies to identify changes in gene expression associated with antigen processing and priming of APC for concurrent antigen presentation to CD4 and CD8 T cells.

## Methods

### Animals

A Holstein steer (2 years old) was used for the blood draw. This steer is part of the Washington State University (WSU) *Map*-free dairy herd. They were held in an open feed lot and managed by the college Animal Resource Unit staff. The collection of blood for *in vitro* experiments was previously approved by Washington State University Institutional Animal Care and Use Committee in association with another study (ASAF 6542).

### Generation of monocyte-derived macrophages

Whole (500 mL) blood was collected from the jugular vein into a bottle containing acid citrate dextrose (ACD) and stored at 4°C until processing. PBMCs were isolated by density gradient centrifugation using Ficoll-Paque™ density 1.077 (GE Healthcare Life Sciences, MA). The isolated PBMCs were then incubated with anti-CD14 magnetic cell sorting microbeads (MACS, Miltenyi Biotec, Germany) and passed through a magnet to collect the CD14+ monocytes. The monocytes were then counted using an automated cell counter (Moxi Z™ OS 4.4, Orflo®, ID) and suspended in culture medium at a concentration of 1 × 10^6^ cells/mL. The culture medium was prepared using DMEM, high glucose, HEPES, no phenol red (Thermo Fisher Scientific, MA) and adding 2.7 mL/250 mL medium of Penicillin-Streptomycin (stock solutions 10,000 U/mL Penicillin and 10,000 µg/mL Streptomycin), 27.5 mL/250 mL medium of heat inactivated calf bovine serum, 500 µL/50 mL β-mercaptoethanol, and glutamine (0.5 mM). The cells were then plated in 6 well plates, 3 mL in each well and 25 ng/mL of GM-CSF was added to each well. The cells were incubated at 37°C with 5% CO_2_.

### Treatments

On Day 6, 1 mL of medium was removed and replaced with fresh warmed medium. One plate was treated with 50 µL of PBS/well, one plate was treated with 5 µg/mL of LPS/well, and one plate was treated with 5 µg/mL of MMP/well. The plates were gently mixed for 45 seconds and then placed back in the incubator for 3 hours.

### RNA isolation

Each well was rinsed twice with 5 mL of PBS. The RNA was isolated using the Aurum™ Total RNA Mini Kit (Bio-Rad Laboratories, CA) procedure for adherent cell cultures and two wells were used for each RNA sample. Briefly, lysis solution was added to the well and pipetted up and down to lyse cells thoroughly, then transferred to a second well and again pipetted up and down to lyse the cells. Next, 70% ethanol was added, mixed, and the mixture was transferred to an RNA binding column and centrifuged (14,000 x g for 1 minute). The column was then washed with low stringency wash solution and centrifuged (14,000 x g for 1 minute). Diluted DNase I was then added directly to the membrane stack at the bottom of each column and incubated for 15 minutes. Afterwards, the column was washed first with high stringency wash solution and centrifuged (14,000 x g for 1 minute) and then with low stringency wash solution and centrifuged (14,000 x g for 1 minute). The column was then centrifuged for an additional 2 minutes (14,000 x g). The RNA binding column was then transferred to a capped microcentrifuge tube and warmed elution solution was added directly to the membrane stack and allowed to sit for 1 minute. Afterwards, the sample was centrifuged for 3 minutes at 8,000 x g to elute the RNA. The quantity and quality of the RNA sample was assessed using a NanoDrop™ One/One^C^ spectrophotometer version 2.4.0.37 (Thermo Fisher Scientific, MA) and the samples were stored at -80°C.

### RNA-sequencing

The samples were sent to Novogene for processing (Sacramento, CA). The quality control for the RNA samples was performed using Qubit and Bioanalyzer instruments. Libraries were then prepared using NEBNext Ultra II non-directional RNA Library Prep kit. Library quality and concentration was assessed with Labchip and quantitative PCR (qPRC). Libraries were sequenced on Novaseq6000 using PE150 sequencing.

### Differential expression analysis on iDEP.93

The read count data from Novogene were uploaded to iDEP.93 (http://bioinformatics.sdstate.edu/idep/) (Ge et al., 2018). Genes with counts per million (CPM) less than 0.5 CPM were filtered out and the data were transformed with EdgeR (log_2_ [CPM +4]). Differential expression (DE) data including log2 fold-change and false discovery rate (FDR)-adjusted p-values were extracted using DESeq2.

### Statistical analysis

The FDR-adjusted p-values extracted from iDEP were calculated using the Benjamini-Hochberg method with an FDR-adjusted p-value threshold of < .05. The expression levels of genes in the MMP-treated MoMΦ versus control MoMΦ and MMP-treated MoMΦ versus LPS-treated MoMΦ were also compared using a two-tailed unpaired t-test. Values of P < .05 were considered significant.

### Phenotyping

The phenotype of the CD14+ cells was assessed at Day 0 right after isolation and on Day 7. To collect the adherent cells on Day 7, the cells were washed once with PBS and then covered with 1 mL 2.5 mM EDTA and refrigerated on ice for 1 hour. The cells were labeled with monoclonal antibodies (mAbs) from the Monoclonal Antibody Center at Washington State University (https://vmp.vetmed.wsu.edu/resources/monoclonal-antibody-center, Pullman, WA). The panel consisted of a negative control, and mAbs specific for CD14 (CAM36A), CD163 (LND68A), CD172a (DH59B), CD209 (209MD26A), and CD205 (ILA53A). All mAbs were developed in mice. Briefly, the cells were washed and then suspended in first wash buffer and divided into 15 mL conical tubes in 100 µL aliquots. The cells were incubated for 15 minutes with 50 µL each of the primary monoclonal antibodies as previously specified (Davis et al., 2007). The cells were washed and then incubated with 100 µL of the goat anti-mouse secondary antibody Alexa Fluor® 488 (Thermo Fisher Scientific, MA) for 15 minutes. The cells were then washed once with second wash buffer, resuspended in 2% PBS buffered formaldehyde, transferred to flow tubes, and stored at 4°C until flow cytometry was performed. Samples were run on a BD FACS Calibur™ and analyzed using FCS Express 7.

## Results

### Phenotype of monocytes following culture for seven days

**Figure 1:**
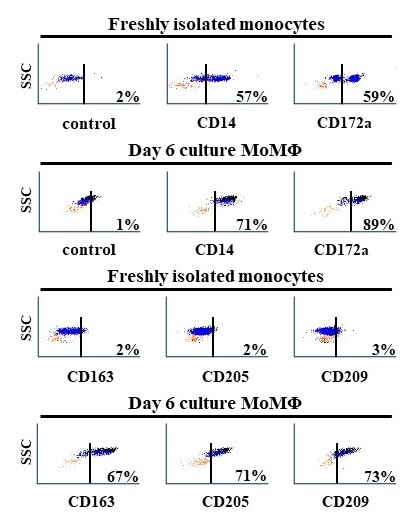
Flow cytometric assay for phenotyping the isolated monocytes on Day 0 and monocyte-derived macrophages (MoMΦ) on Day 6 of culture.

Analysis of the phenotype of freshly isolated monocytes demonstrated they expressed CD14 and CD172a. They were negative for CD163, CD205, and CD209. Following culture for 7 days they expressed all five molecules (Figure 1).

### Comparison of genes differentially expressed following culture of monocytes for seven days

**Figure 2:**
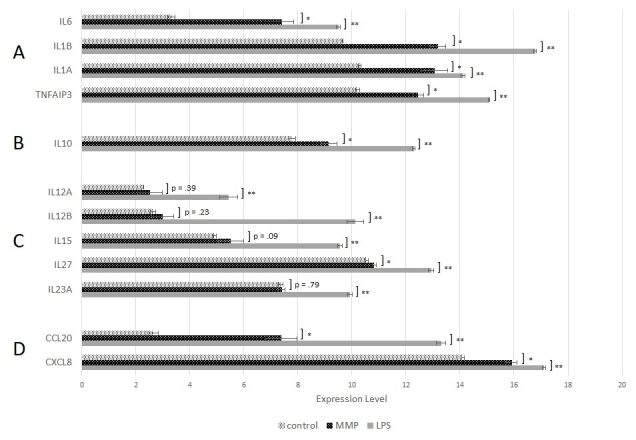
Bar graph representing the mean expression levels of selected genes for control MoMΦ, MMP-treated MoMΦ, and LPS-treated MoMΦ +/- SD. A. Proinflammatory genes; B. Anti-inflammatory genes; C. Proimmune genes; D. Chemokines. P-values were evaluated by unpaired t test (*p-value < .05 for control MoMΦ versus MMP-treated MoMΦ; **p-value < .05 for MMP-treated MoMΦ versus LPS-treated MoMΦ).

The transcriptome was examined for all differentially expressed genes comparing the MoMΦ cultured alone with MMP-treated MoMΦ. 1609 genes were upregulated with an FDR > 0.05 and 1277 downregulated with an FDR < 0.05 three hours after uptake and processing MMP (not shown). For further analysis we selected 10 genes encoding cytokines involved in signaling associated with proinflammatory or inflammatory responses and genes directly involved in signaling to lymphocytes and compared their activation status (Table 1). We also included 2 genes encoding chemokines. LPS upregulated the signal to all the genes selected for analysis in comparison with MoMΦ cultured alone and in the presence of MMP (Table 1 and Figure 2). MMP upregulated the signal to some but not all genes selected for analysis in comparison to the control MoMΦ. The increases were significant but less than the increases observed with LPS. Except for IL-27, differences in expression of genes encoding molecules more directly involved in MoMΦ signaling to CD4 and CD8 T cells were not significantly different than the control.

**Table 1:** Differential expression of MMP-treated MoMΦ versus control MoMΦ for selected genes ranged by fold-change.

**Ensembl ID**	**Gene Symbol**	**Gene name**	**Log2 fold-change**	**FDR-adjusted P value**
ENSBTAG00000021326	CCL20	Chemokine (C-C motif) ligand 20	+6.25	1.04E-26
ENSBTAG00000014921	IL6	Interleukin 6	+4.85	1.99E-36
ENSBTAG00000001321	IL1B	Interleukin 1 beta	+3.56	9.32E-102
ENSBTAG00000010349	IL1A	Interleukin 1 alpha	+2.63	1.36E-78
ENSBTAG00000000436	TNFAIP3	Tumor necrosis factor alpha induced protein 3	+2.22	7.09E-65
ENSBTAG00000019716	CXCL8	Interleukin 8	+1.81	1.13E-49
ENSBTAG00000006685	IL10	Interleukin 10	+1.39	1.07E-10
ENSBTAG00000015150	IL12A	Interleukin 12A (IL-12p35)	+1.189	0.477620
ENSBTAG00000004741	IL12B	Interleukin 12B (IL-12p40)	+0.89	0.360673
ENSBTAG00000018200	IL15	Interleukin 15	+0.72	0.083958
ENSBTAG00000018015	IL27	Interleukin 27	+0.27	0.000148
ENSBTAG00000004378	IL23A	Interleukin 23 subunit alpha	+0.03	0.929378

FDR: false discovery rate; MMP: major membrane protein; IL: interleukin.

## Discussion

Extensive studies have been conducted to elucidate how and when CD4 T cell help is provided to elicit development of CD8 CTL. The *in vivo* and *ex vivo* models used to study this question have not provided an answer (reviewed in Laidlaw et al. (Laidlaw et al., 2016)). The study of the immune response to mycobacterial pathogens in cattle has provided evidence that concurrent tri-directional signaling between APC, CD4 and CD8 T cells is essential for development of CTL (Abdellrazeq et al., 2020a). Comparative studies had demonstrated both blood-derived dendritic cells (bDC) and monocyte-derived MoMΦ primed with a live *Map relA* deletion mutant could be used to elicit a CD8 CTL recall response with autologous PBMC from steers vaccinated with a *relA* mutant (Park et al., 2016). Subsequent follow up studies demonstrated the same recall response could be elicited with a 35 kD membrane peptide, MMP (Abdellrazeq et al., 2018). The requirement for tri-directional signaling was demonstrated with MoDC using an ex vivo platform (Abdellrazeq et al., 2020a). The pilot study described here was conducted to determine if MoMΦ could also be used to extend the studies with RNA-seq to identify the genes associated with antigen processing and priming of APC for antigen presentation to CD4 and CD8 T cells. One steer was used in the pilot study on the assumption that sufficient data could be obtained to answer the question. As reported here, we determined the phenotype of MoMΦ that develop following 7 days of culture. Our previous studies of bovine monocytes, blood DC, MoDC and MoMΦ showed monocytes were negative for CD205 and CD209 and positive for CD14, CD163, and CD172a (Park et al., 2016). Blood DC subsets were positive or negative for CD14, CD163, and CD205. MoDC were positive for CD14, CD172a, CD163, CD205, CD209. MoMΦ were also positive for CD14, CD172a, CD163, CD205, and CD209. In the present study, freshly isolated monocytes were positive for CD14 and CD172a and negative for CD163, CD205 and CD209. MoMΦ cultured for 7 days were positive for CD14, CD172a, CD163, CD205, CD209. CD163 has been reported to be only expressed on anti-inflammatory MoMΦ2 macrophages and not proinflammatory MoMΦ1 in humans (Verreck et al., 2006). CD205 has been shown to be expressed by other leukocyte subsets and is upregulated on DC in humans (reviewed in (Kato et al., 2006)). CD209 has been reported to be expressed only on DC in humans (reviewed in (Geijtenbeek & Gringhuis, 2016; Geijtenbeek et al., 2002)). As in studies of APC in humans and mice, characterization of the phenotype of APCs is still in progress. Studies by Guzman et al. (2019) have provided data showing cultures of monocyte-derived cultures of adherent and nonadherent cells are composed of cells with 2 phenotypes. This heterogeneity was not observed in this study. However, Guzman et al. (2019) used a larger set of mAbs to identify subsets. They were also attempting to define the spectrum of APC phenotypes in cattle. The study was not designed to characterize the phenotype of APC primed for antigen presentation to CD4 and CD8 T cells. Except for expression of CD163, the results obtained on phenotype of antigen primed APC are consistent with our previous studies. MoMΦ have a phenotype similar to MoDC. This suggests they are more similar to DC than conventional MΦ and may account for studies showing they share in the ability to present antigen to CD4 and CD8 T cells. The findings in the present study demonstrate they can be used as one of the cell types for determining the signature of APC primed to present antigen to naïve or memory T cells.

Since it was uncertain as to what the gene signature of MoMΦ, primed with an antigen for 3 hours would be, we examined the entire transcriptome to characterize the signature of an APC primed for antigen presentation to CD4 and CD8 T cells. The finding that a single peptide could be used to prime APC obviated the difficulty of attempting to characterize the signature of an APC primed with whole bacteria (Davis et al., 2021). The use of LPS as a positive control demonstrated that all the genes we selected for analysis were highly upregulated 3 hours after culture with LPS. In contrast, only the sets of genes associated with modulating the inflammatory or anti-inflammatory response were upregulated. The gene encoding the chemokines selected for analysis were also upregulated. Except for IL-27, all the genes directly involved in signaling to CD4 and CD8 were not upregulated. This was an unexpected but important observation that needs to be duplicated. Extensive studies have shown that IL-12, IL-23, and IL-27 are directly involved in signaling to CD4 and CD8 T cells and their subsequent differentiation into cells with effector or regulatory activity. Our previous studies demonstrated that APC primed for 3 hours elicit development of CD8 CTL. The current observations suggest antigen primed APC require contact and signals from CD4 and CD8 before IL-12 or IL-23 and IL-27 genes are activated for secretion of their respective cytokines.

## Conclusion

In summary, the present studies have shown that MoMΦ can be included along with primary blood DC and MoDC in studies designed to begin characterizing the tri-directional signaling required for development of CTL. Although APC primed for antigen presentation for 3 hours are appropriate for analysis of the rapid signaling that must occur on contact with CD4 and CD8 T cells, the time is too late for characterizing the signaling that takes place at the time of uptake and processing of antigen for concurrent presentation of antigen derive peptides through MHC class I and class II molecules. A study by Zieglar and Unanue showed processing occurs within minutes following uptake of an antigen (Ziegler & Unanue, 1981). Sequential samples will be required to capture the events of antigen uptake and processing. Likewise, sequential sampling will be required to capture the tri-directional signaling events that lead to development of primary and recall CD8 CTL responses. The identification of a single peptide that elicits a CTL response overcomes some of the difficulties associated with using RNA-seq to characterize the immune response. The use of a single steer was sufficient for obtaining data needed for more in-depth analysis.
